# Effect of rapid methicillin-resistant *Staphylococcus aureus* nasal polymerase chain reaction screening on vancomycin use in the intensive care unit

**DOI:** 10.1093/ajhp/zxab296

**Published:** 2021-07-23

**Authors:** Calvin Diep, Lina Meng, Samaneh Pourali, Matthew M Hitchcock, William Alegria, Rebecca Swayngim, Ran Ran, Niaz Banaei, Stan Deresinski, Marisa Holubar

**Affiliations:** 1 Department of Pharmacy, Stanford Healthcare, Palo Alto, CA, USA; 2 Department of Infectious Diseases, Central Virginia VA Health Care System, Richmond, VA, USA; 3 Department of Pharmacy, Denver Health Medical Center, Denver, CO, USA; 4 Department of Critical Care Medicine, Oregon Health & Science University, Portland, OR, USA; 5 Division of Infectious Diseases and Geographic Medicine, Stanford University School of Medicine, Palo Alto, CA; 6 Department of Pathology, Stanford University School of Medicine, Palo Alto, CA, USA; 7 Division of Infectious Diseases and Geographic Medicine, Stanford University School of Medicine, Palo Alto, CA, USA

**Keywords:** antimicrobial stewardship, intensive care unit, methicillin-resistant *Staphylococcus aureus*, pneumonia, rapid diagnostics, vancomycin

## Abstract

**Purpose:**

To determine the impact of a pharmacist-driven methicillin-resistant *Staphylococcus aureus* (MRSA) nasal polymerase chain reaction (PCR) screen on vancomycin duration in critically ill patients with suspected pneumonia.

**Methods:**

This was a retrospective, quasi-experimental study at a 613-bed academic medical center with 67 intensive care beds. Adult patients admitted to the intensive care unit (ICU) between 2017 and 2019 for 24 hours or longer and empirically started on intravenous vancomycin for pneumonia were included. The primary intervention was the implementation of a MRSA nasal PCR screen protocol. The primary outcome was duration of empiric vancomycin therapy. Secondary outcomes included the rate of acute kidney injury (AKI), the number of vancomycin levels obtained, the rate of resumption of vancomycin for treatment of pneumonia, ICU length of stay, hospital length of stay, the rate of ICU readmission, and the rate of in-hospital mortality.

**Results:**

A total of 418 patients were included in the final analysis. The median vancomycin duration was 2.59 days in the preprotocol group and 1.44 days in the postprotocol group, a reduction of approximately 1.00 day (*P* < 0.01). There were significantly fewer vancomycin levels measured in the postprotocol group than in the preprotocol group. Secondary outcomes were similar between the 2 groups, except that there was a lower rate of AKI and fewer vancomycin levels obtained in the postprotocol group (despite implementation of area under the curve–based vancomycin dosing) as compared to the preprotocol group.

**Conclusion:**

The implementation of a pharmacist-driven MRSA nasal PCR screen was associated with a decrease in vancomycin duration and the number of vancomycin levels obtained in critically ill patients with suspected pneumonia.

**Key Points**

- A retrospective, quasi-experimental study was conducted to assess the impact of a pharmacist-driven methicillin-resistant *Staphylococcus aureus* (MRSA) nasal polymerase chain reaction (PCR) screening protocol on vancomycin use in the intensive care unit.
- The protocol was associated with reduced vancomycin duration of approximately 1 day and avoidance of measurement of serum levels, with no vancomycin levels obtained in 19% of patients before vs 53% of patients after implementation.
- Use of the MRSA PCR screen was associated with safe de-escalation of vancomycin therapy without increased clinical adverse events in a diverse critically ill population.

Vancomycin is commonly prescribed for empiric coverage of methicillin-resistant *Staphylococcus aureus* (MRSA) pneumonia in the intensive care unit (ICU).^1^ National guidelines for the management of community-acquired pneumonia (CAP) and hospital-acquired pneumonia (HAP) recommend empiric MRSA coverage in patients with specific risk factors or residing in areas with high MRSA prevalence.^2,3^ However, recently, there have been concerns about vancomycin overuse, disproportionate to the relative rarity of MRSA pneumonia, which may be associated with increased adverse events and cost.^4^ For this reason, vancomycin de-escalation has become an attractive target for antimicrobial stewardship programs.

The MRSA nasal polymerase chain reaction (PCR) screen is a rapid surveillance tool used to detect MRSA colonization of the anterior nares, which has been shown to be a risk factor for clinical MRSA respiratory infection. Prior studies and meta-analyses have reported a negative predictive value (NPV) of greater than 98% and have demonstrated safe de-escalation of anti-MRSA therapy with negative MRSA nasal PCR results.^5-10^ In addition, pharmacy-driven protocols allowing pharmacists to order this assay have been shown to decrease vancomycin duration and the number of vancomycin levels obtained in the inpatient setting.^11-15^ The most recent Infectious Diseases Society of America and American Thoracic Society guideline for the management of CAP also supports utilization of the MRSA nasal PCR screen as an antibiotic de-escalation tool.^2^ Although prior studies have shown a high NPV for the MRSA nasal PCR screen in a heterogenous patient population, including in patients requiring ICU-level care, there are limited data describing the performance of this screen in subsets of different types of pneumonia, particularly in critically ill patients with HAP and ventilator-associated pneumonia (VAP). Local MRSA prevalence rates are one factor that impacts test performance. At our institution in 2018, MRSA prevalence was 22% (ICU, 29%) from all sources and 33% (ICU, 36%) from respiratory cultures.^16^

Since 2010, Stanford Health Care (SHC) has performed routine MRSA screening via culture (turnaround time of 2 days, nursing-driven protocol) of the anterior nares of all inpatients admitted to the ICU as part of a state mandate.^17^ In May 2018, SHC initiated an independent, pharmacist-driven testing protocol authorizing pharmacists to order MRSA nasal PCR screens (turnaround time of 4-6 hours) for all inpatients empirically started on anti-MRSA therapy (ie, linezolid or vancomycin) for suspected pneumonia. The aim of this study was to assess the impact and safety of the pharmacist-driven MRSA nasal PCR protocol in critically ill patients at a center with a MRSA prevalence of 22%. Our hypothesis was that use of the MRSA nasal PCR screen would lead to shorter durations of vancomycin therapy given that the PCR test results can be obtained within 6 hours, allowing for quicker de-escalation of vancomycin therapy. Furthermore, the impact of the MRSA nasal PCR screen on vancomycin use in prespecified subsets of patients, including those with underlying immunosuppression and those requiring mechanical ventilation, vasopressor support, and/or extracorporeal membrane oxygenation (ECMO), was evaluated.

## Methods

This was a single-center, retrospective, quasi-experimental study comparing outcomes before and after protocol implementation at a 613-bed academic medical center. The time period before protocol implementation ranged from January 1, 2017, to June 1, 2017, while the time period following protocol implementation was from September 3, 2018, to September 3, 2019. We included a time gap between the pre- and postprotocol periods to allow for practice adjustments following protocol implementation and a switch to area under the curve (AUC) monitoring at our institution. Patients were included if they were 18 years of age or older, were admitted to the ICU for 24 hours or longer, and were empirically started on vancomycin therapy for pneumonia. Exclusion criteria included receipt of vancomycin therapy for indications other than pneumonia and a past medical history of cystic fibrosis (based on provider group preference).

Under the pharmacy-driven protocol, pharmacists entered a MRSA nasal PCR order upon receipt of an intravenous vancomycin order with an indication for empiric pneumonia, specified by the ordering provider in the electronic medical record (EMR) order, 7 days a week. De-escalation of anti-MRSA therapy is encouraged by hospital guidelines for negative results if pneumonia is presumed to be the source of infection.^18,19^ Team-based pharmacists communicated negative results during team rounds. In both time periods, team-based clinical pharmacists participated daily in clinical rounds from Monday through Friday. Starting in February 2019, infectious disease pharmacists communicated negative PCR results to the surgical ICU via stewardship handshake rounds twice weekly. Ultimately, discontinuation was at the discretion of the primary medical team. The MRSA PCR screen was not ordered for patients with an existing MRSA nasal PCR test within the last 7 days, with confirmed MRSA in respiratory cultures in the last 7 days, or receiving vancomycin for prophylaxis after transplantation (see eAppendix for full protocol). Samples for MRSA nasal PCR and nasal culture were collected as a nasal swab by nurses and analyzed 24 hours a day, 7 days a week, using MRSASelect (Bio-Rad Laboratories, Hercules, CA) and the Gen Xpert MRSA assay (Cepheid, Sunnyvale, CA), respectively, at the Stanford clinical laboratory.^20^

In June 2018, there was an institution-wide switch from vancomycin trough monitoring to AUC monitoring.^21-24^ In the preprotocol group, vancomycin was monitored using single-level trough concentrations, while in the postprotocol group vancomycin was monitored using the peak and trough levels to calculate an AUC by the trapezoidal method. Levels were typically determined after at least the third dose to estimate steady-state levels. Pharmacist oversight of vancomycin use, monitoring, and dosing was unchanged with this switch in monitoring method. There were also no significant changes in antimicrobial stewardship services during the study period.

Data were extracted from the EMR based on orders for vancomycin per pharmacy with an indication for empiric pneumonia coverage for the preprotocol group and an order for a MRSA nasal PCR test for the postprotocol group. The study was approved by the SHC institutional review board, and data collection was performed by review of the EMR. The following information was collected for patients who met the prespecified inclusion criteria: baseline demographics, comorbidities, pneumonia classification, MRSA nasal culture results in the preprotocol group, MRSA PCR results in the postprotocol group, presence of imaging suggestive of pneumonia, and respiratory microbiology culture results. In addition, data on use of ECMO, renal replacement therapy, mechanical ventilation, and/or vasopressors at the time of vancomycin initiation were also collected. Immunocompromise was defined as a history of solid organ transplantation, bone marrow transplantation, use of steroids at doses of 20 mg of prednisone equivalents or greater for more than 14 days, receipt of a biological agent in the preceding 30 days, chemotherapy within the preceding 6 months, and/or human immunodeficiency virus infection with a CD4^+^ T cell count of 200 cells/mL or less. Patients were designated as having CAP if vancomycin therapy was started 48 hours or earlier from hospital admission and as having HAP or VAP if vancomycin therapy was started more than 48 hours after hospital admission. These classifications were cross-referenced with provider notes in the EMR to verify that symptoms were present. Acute kidney injury (AKI) was defined as an increase in serum creatinine levels of more than 0.5 mg/dL or 2 times baseline within 7 days of initiation of vancomycin therapy, as defined by the RIFLE criteria of “injury” or worse.^25^ Patients on dialysis at the time of vancomycin initiation were not included in the AKI analysis, as this outcome could not be assessed. Imaging suggestive of pneumonia was defined based on the presence of a new consolidation or infiltrate on chest imaging documented in the radiological report, as determined by the interpreting radiologist.

The primary outcome was duration of vancomycin therapy in days before and after protocol implementation. Duration of therapy was calculated based on the start and stop times of vancomycin per the pharmacy’s orders placed in the EMR. Secondary outcomes included the rate of AKI, the number of vancomycin levels obtained per patient (trough and random levels), the rate of vancomycin reinitiation within 3 and 7 days of discontinuation for empiric treatment of pneumonia, ICU length of stay, hospital length of stay, the rate of ICU readmission due to pneumonia, and the in-hospital infection-related mortality rate. Infection-related mortality was assessed based on the cause of death documented in the EMR. Duration of vancomycin therapy in population subgroups, including immunocompromised patients, those receiving mechanical ventilation, those on vasopressors, and those on ECMO, was also evaluated.

On the basis of the study by Willis et al^11^ with a median vancomycin duration of 4.2 days (interquartile range, 2.8-5.8 days) in the preimplementation arm, we estimated that 78 patients would be needed in each group (156 in total) to achieve 80% power to detect a 1-day reduction in vancomycin therapy assuming a 2-sided *α* value of 0.05. The sample size was inflated by 15% to a minimum of 89 patients in each group (178 in total) to ensure adequate power, given that the duration of vancomycin therapy could not be assumed to have a normal distribution. IBM SPSS Statistics version 22 (IBM Analytics, Armonk, NY) was used to perform all statistical analyses with a predefined significance level of 0.05 by 2-tailed asymptotic or exact tests. Nonparametric continuous variables were analyzed using Mann-Whitney *U* tests, and categorical variables were analyzed using Pearson *χ*^2^ or Fisher’s exact tests. Parametric continuous variables were compared using ANOVA. The interactions between parametric continuous variables were explored with generalized linear models or MANOVA.

## Results

**Figure 1.**
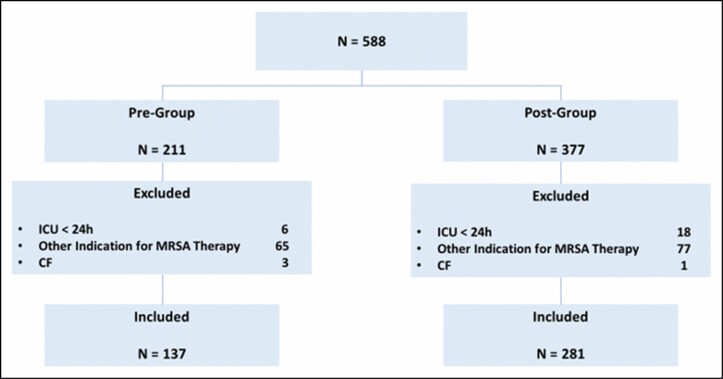
Flowchart of patient selection. CF indicates cystic fibrosis; ICU, intensive care unit; MRSA, methicillin-resistant *Staphylococcus aureus*; pre-group, preprotocol group; post-group, postprotocol group.

A total of 588 patients were screened for inclusion. Of these patients, 418 were included in the final analysis, 137 in the preprotocol group and 281 in the postprotocol group. The most common reason for exclusion was use of vancomycin for indications other than pneumonia (*n* = 142). Details for patient inclusion and exclusion are shown in Figure 1. Although baseline characteristics were generally similar between the groups (Table 1), patients in the postprotocol group were more likely to require vasopressors at the time of vancomycin initiation (46% vs 32%, *P* = 0.01).

**Table 1.** Baseline Characteristics

Characteristic	Preprotocol Group (*n* = 137)	Postprotocol Group (*n* = 281)	*P* Value
Age, median (IQR), years	63 (50-73)	63 (52-74)	0.57
Male sex, No. (%)	93 (68)	182 (65)	0.58
Comorbidities, No. (%)			
Diabetes	33 (24)	60 (21)	0.53
Chronic kidney disease	22 (16)	65 (23)	0.10
Respiratory illness	19 (14)	55(20)	0.15
Immunocompromise	20 (15)	70 (25)	0.08
Solid organ transplantation	9 (7)	23 (8)	
Bone marrow transplantation	2 (2)	12 (4)	
Other^a^	9 (7)	35 (12.5)	
Extracorporeal membrane oxygenation, No. (%)	16 (12)	18 (6)	0.06
Renal replacement therapy, No. (%)	9 (7)	32 (11)	0.25
Continuous renal replacement therapy	7 (5)	21 (7)	
Intermittent hemodialysis	2 (1)	11 (4)	
Mechanical ventilation, No. (%)	83 (61)	177 (63)	0.63
Vasopressor use, No. (%)	44 (32)	129 (46)	0.01

Abbreviation: IQR, interquartile range.

^a^Use of steroids at doses of 20 mg of prednisone equivalents or greater for more than 14 days, receipt of a biological agent in the preceding 30 days, chemotherapy within the preceding 6 months, or human immunodeficiency virus with a CD4^+^ T cell count of 200 cells/mL or less.

Pneumonia classification was similar between the pre- and postprotocol groups, with 181 patients designated as having CAP (54 [39%] and 127 [45%], respectively) and 237 patients designated as having HAP or VAP (83 [61%] and 154 [55%], respectively; Table 2). MRSA nasal screening by culture upon admission to the ICU was negative in 89% (105/119) of patients in the preprotocol group, and MRSA PCR results were negative in 93% (262/281) of patients in the postprotocol group. MRSA was isolated from respiratory cultures in a similar proportion of patients in the 2 groups (4% in the preprotocol group and 3% in the postprotocol group). The percentage of MRSA positivity in patients with positive respiratory cultures was 16% (6/37) vs 8% (7/85) in the pre- vs postprotocol group. All baseline microbiology characteristics are shown in Table 2.

**Table 2.** Baseline Microbiology Characteristics

Characteristic	Preprotocol Group (*n* = 137)	Postprotocol Group (*n* = 281)	*P* Value
Pneumonia classification, No. (%)			0.26
CAP	54 (39)	127 (45)	
HAP or VAP	83 (61)	154 (55)	
Imaging suggesting pneumonia, No. (%)	69 (50)	153 (54)	0.43
Respiratory culture result, No. (%)	104 (76)	175 (62)	0.01
Enterobacteriaceae^a^	11 (8)	26 (9)	
*Haemophilus influenzae*	1 (1)	5 (2)	
MSSA	9 (7)	14 (5)	
MRSA	6 (4)	7 (3)	
*Pseudomonas*	1 (1)	15 (5)	
Other^b^	9 (7)	18 (6)	
Normal flora/no growth	67 (49)	90 (32)	
Respiratory culture result not available, No. (%)	33 (24)	106 (38)	

Abbreviations: CAP, community-acquired pneumonia; HAP, hospital-acquired pneumonia; MRSA, methicillin-resistant *Staphylococcus aureus*; MSSA, methicillin-susceptible *Staphylococcus aureus*; VAP, ventilator-associated pneumonia.

^a^Enterobacteriaceae included *Escherichia coli* and *Klebsiella*, *Enterobacter*, and *Serratia* species.

^b^Other included *Stenotrophomonas* and *Streptococcus* species.

In the postprotocol group, MRSA nasal PCR was negative in 262 (93%) patients, of whom none had MRSA isolated in respiratory culture. Of the 19 patients who had a positive MRSA nasal PCR result, 7 had MRSA isolated on their respiratory culture. The calculated sensitivity, specificity, positive predictive value (PPV), and NPV for the MRSA nasal PCR screen were 100%, 95.6%, 36.8%, and 100%, respectively.

**Figure 2.**
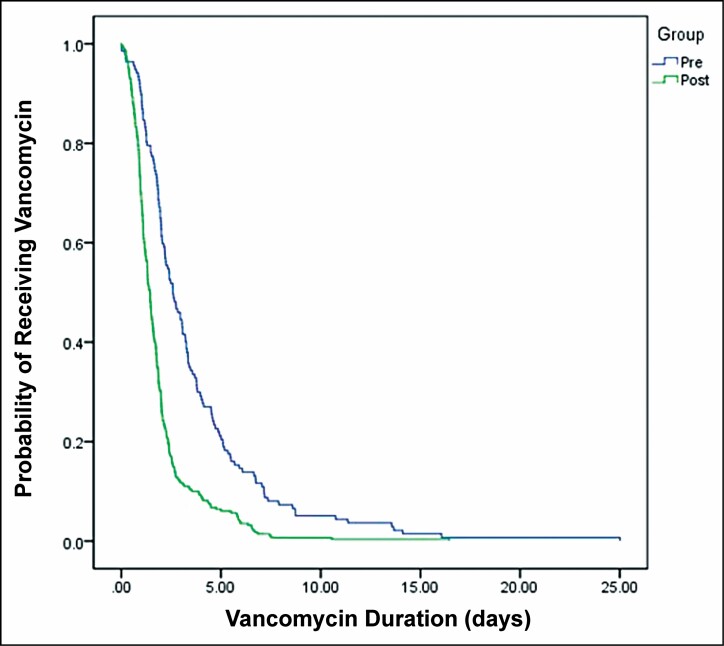
Vancomycin duration by study group. Pre indicates preprotocol group; post, postprotocol group.

The median vancomycin duration was 2.59 days in the preprotocol group and 1.44 days in the postprotocol group (*P* < 0.01; Table 3 and Figure 2). In patients receiving ECMO, on mechanical ventilation, or receiving vasopressors, the median vancomycin duration was significantly shorter in the postprotocol group than in the preprotocol group (Table 3). There was no significant difference in median vancomycin duration between the 2 groups in immunocompromised patients (Table 3).

**Table 3.** Primary and Secondary Outcomes

Outcome	Preprotocol Group (*n* = 137)	Postprotocol Group (*n* = 281)	*P* Value
Primary outcome			
Vancomycin duration, median (IQR), days	2.59 (1.68-4.55)	1.44 (0.91-2.08	<0.01
Secondary outcomes			
Vancomycin duration, median (IQR), days			
Extracorporeal membrane oxygenation (34 patients)	3.78 (2.17-7.66)	1.76 (1.02-2.39)	<0.01
Immunocompromise (90 patients)	2.50 (1.92-3.30)	1.73 (0.93-2.84)	0.26
Mechanical ventilation (124 patients)	2.48 (1.67-4.59)	1.55 (0.97-2.27)	<0.01
Vasopressors (78 patients)	2.68 (1.71-5.23)	1.35 (0.89-2.23)	<0.01
Hospital length of stay, median (IQR), days	19.3 (12.3-33.9)	16.1 (8.9-31.6)	0.09
ICU length of stay, median (IQR), days	9.8 (4.76-18.67)	8.25 (3.96-17.94)	0.23
In-hospital mortality, No. (%)	43 (31)	62 (23)	0.06
ICU readmission due to pneumonia, No. (%)	1 (1)	4 (1.4)	0.54
Rate of acute kidney injury, No. (%)^a,b^	31 (24)	33 (13)	0.01
Resumption of vancomycin at 3 days, No. (%)	9 (6.6)	22 (8)	0.65
Resumption of vancomycin at 7 days, No. (%)	21 (15)	44 (16)	0.93
Vancomycin levels (random or trough) obtained per patient, median	1	0	<0.01
Trough	1	0	
Random	0	0	
No levels (random or trough) obtained, No. (%)	26 (19)	149 (53)	<0.01

Abbreviations: ICU, intensive care unit; IQR, interquartile range.

^a^Defined as a serum creatinine level above 0.5 mg/dL or an increase in serum creatinine levels of more than 2-fold from baseline within 7 days of initiation of vancomycin therapy per the RIFLE criteria.

^b^A total of 377 patients were included in the analysis (128 in the preprotocol group and 277 in the postprotocol group).

A total of 377 patients were included in the AKI analysis, 128 in the preprotocol group and 249 in the postprotocol group. The rate of AKI was 24% in the preprotocol group and 13% in the postprotocol group (*P* = 0.01). The median number of vancomycin levels (random and trough) obtained per patient was 1 in the preprotocol group and 0 in the postprotocol group (*P* < 0.01). No levels were obtained in 19% of patients in the preprotocol group as compared to 53% of patients in the postprotocol group (*P* < 0.01). There were no statistically significant differences in the other secondary outcomes, including hospital length of stay, ICU length of stay, in-hospital mortality rate, rate of ICU readmission due to pneumonia, and rates of resumption of vancomycin therapy within 3 and 7 days. Hospital length of stay, ICU length of stay, and in-hospital mortality rate were not significantly different between the groups for patients receiving ECMO, mechanical ventilation, or vasopressors (data not shown). Data for all primary and secondary outcomes are shown in Table 3.

## Discussion

In this study including a diverse critically ill patient population, the implementation of a pharmacy-driven MRSA PCR protocol was associated with a reduction in vancomycin duration of approximately 1.00 day as well as a reduction in the number of vancomycin levels obtained despite a concurrent switch from trough-based to AUC-based vancomycin monitoring. The incidence of AKI was also reduced following implementation of the protocol; however, this may have been confounded by the switch to AUC-based vancomycin monitoring in the postprotocol group. This study reaffirmed the clinical utility of the MRSA PCR screen as a de-escalation tool, demonstrating a PPV of 36.8% and an NPV of 100%, in line with prior studies conducted in general ward and ICU patients.^7-9^

Previous studies have evaluated the role of MRSA nasal PCR screening in reducing vancomycin duration.^11-15^ However, only 15% to 37% of the patient populations in these studies were critically ill. Smith et al^5^ reported an NPV of 99% in 400 critically ill patients who had respiratory cultures performed within 7 days of a nasal PCR result. However, the study did not report clinical outcomes and had a low compliance rate for de-escalation within 24 hours of a negative PCR result (45%). In contrast, our compliance rate was 68%. In addition, there are limited data regarding the reliability of the MRSA nasal PCR screen in patients receiving mechanical ventilation, on ECMO, or who are immunocompromised. Reassuringly, there were no false-negative results observed in this study, even with inclusion of these subsets of critically ill patients.

Rapid diagnostic tools such as the MRSA nasal PCR screen can play a vital role in antimicrobial stewardship efforts, even in critically ill patients requiring higher levels of hemodynamic support. Antibiotic de-escalation in the ICU can be challenging, especially in patients with severe sepsis requiring mechanical and pharmacological support to maintain adequate cardiopulmonary function. Limited published data on the impact of the MRSA nasal PCR screen in patients requiring mechanical ventilation, vasopressors, and/or ECMO exist. In this study, vancomycin duration was significantly shorter after the implementation of MRSA nasal PCR screening in the subgroup of patients receiving ECMO. There were only 34 patients in this study who received ECMO, but, to our knowledge, this is the first study to describe use of the MRSA nasal PCR screen in this patient population. In addition, vancomycin duration was shorter in the postprotocol group despite significantly more patients receiving vasopressors at the time of vancomycin initiation as compared to the preprotocol group. In the subset of patients receiving mechanical ventilation and vasopressors, vancomycin duration was also significantly shorter in the postprotocol group than in the preprotocol group. In addition, implementation of the MRSA nasal PCR screen was not associated with increased clinical adverse outcomes, as no differences in secondary outcomes were observed in these patient subgroups before and after implementation. Despite the reluctance to de-escalate in the ICU, we found that use of the MRSA PCR screen can lead to safe de-escalation of vancomycin therapy used for empiric pneumonia treatment in this setting.

Immunocompromised patients are another population where antibiotic de-escalation is challenging. Factors that may make de-escalation difficult include prolonged neutropenia, persistent fevers of unknown origin, an unclear source of infection, previous exposure to a prolonged course of antibiotics, high mortality risk, and limited evidence to guide practice.^26-28^ In this study, vancomycin duration was approximately 2-fold shorter in immunocompromised patients in the postprotocol group as compared to the preprotocol group, yet this difference was not significant. The lack of a statistically significant difference in vancomycin duration with use of the MRSA nasal PCR screen in this population may reflect some of the complexities with immunocompromised patients as discussed above. However, vancomycin therapy was discontinued within 24 hours of a negative MRSA nasal PCR result in 70% of immunocompromised patients in this study.

We found that the MRSA nasal PCR screen was associated not only with reduced overall vancomycin duration but also with a decreased incidence of AKI. Nephrotoxicity is a widely recognized adverse effect of vancomycin therapy despite close therapeutic monitoring. The incidence of vancomycin-induced AKI ranges from 5% to 43% across various studies, while the relative risk of AKI has been reported as 2.45.^21,22^ In the ICU setting, the rate of vancomycin-induced AKI is even more unclear as these patients have multiple risk factors and often receive concomitant nephrotoxic medications. Baby et al^12^ found that the incidence of AKI decreased from 26% to 3% with use of the MRSA nasal PCR screen, while Willis et al^11^ did not find a significant reduction. The mixed results regarding reduction in AKI incidence across prior studies may be attributed to the different patient populations studied. In this study, the reduction in incidence of new AKI seen may have been associated with reduced vancomycin exposure secondary to implementation of the MRSA nasal PCR screen. These results, however, may have been confounded by an institution-wide switch from vancomycin trough monitoring to AUC-based monitoring in June 2018, which may have contributed to the decreased rate of nephrotoxicity.^23,24^

In this study, the implementation of a pharmacist-driven MRSA nasal PCR protocol was associated with fewer vancomycin levels obtained. With recent guidelines recommending AUC-based targets for vancomycin monitoring,^22^ the need to obtain additional vancomycin levels may increase for those switching from trough- to AUC-based estimates via 2-level measurements and the trapezoidal rule. The rapid result time of the MRSA PCR screen can lead to discontinuation of vancomycin therapy before the need to assess drug levels and thereby offset some of the anticipated increase in laboratory workload. In this study, approximately 50% of patients in the postprotocol group had no vancomycin levels determined as compared to only 19% in the preprotocol group. The MRSA PCR screen can be a useful tool to decrease the number of vancomycin levels obtained as well as the labor required to time and measure levels.

There were several limitations to our study, including its single-center, retrospective nature and the potential confounders inherent to such a design. The number of patients was imbalanced in the preprotocol and postprotocol groups. Sensitivity analysis showed similar primary outcomes when using matched months in the postprotocol and preprotocol groups. Median vancomycin duration was 2.59 days (preprotocol group) vs 1.46 days (matched postprotocol group) and 1.44 days (full postprotocol group). At baseline, the postprotocol group had a larger proportion of patients on vasopressors at the start of vancomycin therapy. Analysis with a generalized linear model was also performed and showed that vasopressor use was not associated with vancomycin duration nor was its interaction with the pre- and postprotocol groups associated with significant differences in vancomycin duration. We did not include prior anti-MRSA therapy or nasal decolonization, which may have impacted the sensitivity of nasal cultures more than that of nasal PCR. However, we did not find false-negative PCR results based on respiratory cultures (NPV, 100%). Data on concomitant use of nephrotoxic mediations were not collected, thus limiting our assessment of AKI. Another limitation was that this study did not investigate the effects of the MRSA nasal PCR screen on duration of therapy with linezolid, another agent used to empirically treat MRSA pneumonia. However, empiric linezolid use for pneumonia at SHC was low and stable throughout the study period without an increase in usage with introduction of the MRSA nasal PCR screen. In addition, the proportion of patients receiving ECMO, mechanical ventilation, or vasopressors or who were immunocompromised was low in this study, representing approximately 5%, 30%, 19%, and 20% of the patient population, respectively. More data are needed in these patient populations. MRSA prevalence in the ICU was 28.6% (all sources) and 36% (respiratory sources) in SHC ICUs during the study period. Previous studies have reported that MRSA accounts for 20% to 40% of patients with HAP or VAP.^29^ The results of this study may not be generalizable to institutions with higher MRSA prevalence.

## Conclusion

This study, which focused specifically on critically ill patients and subsets of patients commonly encountered in the ICU, showed that a pharmacist-driven MRSA nasal PCR screen was associated with a reduction in vancomycin duration of approximately 1 day without an increase in adverse outcomes at a large academic medical center. This study supports the utility and efficacy of the MRSA nasal PCR screen to guide vancomycin de-escalation in a diverse population of critically ill patients with suspected pneumonia. Further studies are needed to confirm the safety and utility of this test in patients receiving ECMO, mechanical ventilation, or vasopressors and in immunocompromised patients.

## Supplementary Material

zxab296_suppl_Supplementary_Materials
