# Supplementary material for: Effect of rapid methicillin-resistant Staphylococcus aureus nasal polymerase chain reaction screening on vancomycin use in the intensive care unit
Source: Am J Health Syst Pharm. 2021 Jul 23;78(24):2236–44. doi: 10.1093/ajhp/zxab296 (PMC8661079; doi:10.1093/ajhp/zxab296)
Supplement: zxab296_suppl_Supplementary_Materials [file zxab296_suppl_supplementary_materials.docx]

**eAppendix**

**MRSA nasal PCR sensitivity, specificity, PPV, NPV calculations (All patients)**

|  | MRSA isolated in culture | MRSA not isolated in culture | Total |
| --- | --- | --- | --- |
| MRSA PCR screen (+) | 7 **[A]** | 12 **[B]** | 19 **[A+B]** |
| MRSA PCR screen (-) | 0 **[C]** | 262 **[D]** | 262 **[C+D]** |
| Total | 7 **[A+C]** | 274 **[B+D]** | 281 |

| Sensitivity: A/(A+C) x 100 | 7/7 | 100% |
| --- | --- | --- |
| Specificity: D/B+D) x 100 | 262/274 | 95.6% |
| PPV: A/(A+B) x 100 | 7/19 | 36.8% |
| NPV: D/(C+D) x 100 | 262/262 | 100% |

**SHC Pharmacist-driven MRSA nasal PCR screening protocol (v.10/2017)**

POLICY

SHC will utilize nasal MRSA PCR screening to guide de-escalation of unnecessary empiric coverage of MRSA pneumonia. Pharmacists will:

1. Order nasal MRSA PCR for vancomycin or linezolid initiated empirically for suspected MRSA pneumonia

Exclusions

1. Existing nasal MRSA PCR test performed within last 7 days
2. Confirmed MRSA in respiratory culture or nasal culture in the last 7 days (If nasal MRSA screen by culture is pending or negative, pharmacist should still order a nasal PCR. See FAQ on ASP intranet page)
3. CF patients and lung transplant post-op prophylaxis (due to provider group preference)

PROCEDURES

1. Pharmacist Responsibility
2. Order nasal MRSA screen by PCR for new Vancomycin-Per-Pharmacy or linezolid orders with indication stated as “pulmonary” (e.g. pneumonia)
3. See above for exclusions
4. Orders will be entered using the “Per Protocol without cosign” mode
5. Provider Responsibility
6. Follow up on nasal MRSA PCR and de-escalate vancomycin or linezolid if negative and clinically appropriate
